# Dynamic Hydrogels: Adaptive Biomaterials for Engineering Tumor Microenvironment and Cancer Treatment

**DOI:** 10.3390/ijms26199502

**Published:** 2025-09-28

**Authors:** Yuting Wu, Yifei Xiao, Bohan Yin, Siu Hong Dexter Wong

**Affiliations:** 1 School of Medicine and Pharmacy, The Ocean University of China, Qingdao 266100, China; 2 Laboratory for Marine Drugs and Bioproducts, Qingdao Marine Science and Technology Center, Qingdao 266237, China

**Keywords:** dynamic hydrogels, tumor microenvironment engineering, stimuli-responsive materials, precision drug delivery, translational challenges

## Abstract

Dynamic hydrogels are revolutionizing tumor microenvironment (TME) engineering through their stimuli-responsive adaptability, mechanical tunability, and capacity for multifunctional integration. In addition, they are excellent biomaterials for cancer treatments, including their biomimetic properties and controlled cargo release capability. This review introduces the rational design and principles of dynamic hydrogels for recreating the tumor microenvironment and cancer therapy, including natural/synthetic hydrogels, multi-stimuli responsive hydrogels, and multi-drug loading hydrogels. These designs emphasize their unique roles in overcoming drug resistance, enhancing immunotherapy, and enabling patient-specific models. We highlight breakthroughs such as dual-responsive nanocomposites and microfluidic-integrated 3D platforms while addressing translational hurdles like cytotoxicity and regulatory delays. By proposing strategies to bridge material science with clinical needs, this work positions dynamic hydrogels as pivotal tools for next-generation precision oncology.

## 1. Introduction

**Figure 1:**
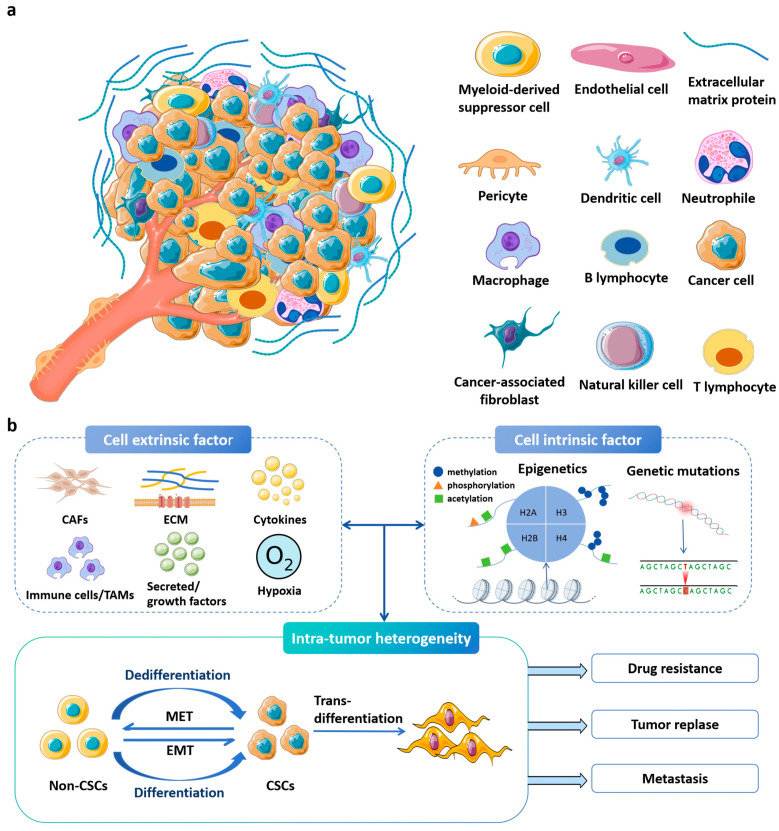
The highly complex and dynamic tumor microenvironment. (**a**) Tumor microenvironment. Various host cells and proteins work together to support cancer cell survival and progression. The image was generated by using BioRender.com. Adapted from [1] with modifications. (**b**) Tumor heterogeneity is primarily driven by both cellular and non-cellular factors, encompassing genetic and epigenetic alterations in cancer cells as well as disturbances in the microenvironment. Cancer stem cells (CSCs) exhibit an induced epithelial–mesenchymal transition (EMT) system, typically existing in an intermediate state. This process relies on genetic mutations, epigenetic alterations, and transcriptional modifications in cancer cells, as well as signals provided by the tumor microenvironment (TME), such as cancer-associated fibroblasts (CAFs), tumor-associated macrophages (TAMs), immune cells, extracellular matrix (ECM), cytokines, and secreted or growth factors. Therefore, intratumoral heterogeneity may play a crucial role in the development of effective treatment strategies for drug resistance, tumor recurrence, and metastasis [2]. Abbreviations: CSCs, cancer stem cells; EMT, epithelial-to-mesenchymal transition; MET, mesenchymal-to-epithelial transition; ECs, endothelial-like cells; CAFs, cancer-associated fibroblasts; TAMs, tumor-associated macrophages; ECM, extracellular matrix.

The tumor microenvironment (TME) of solid tumors is a highly complex and dynamic entity consisting of (1) specific cells (e.g., tumor cells, stromal cells, immune cells), (2) extracellular matrix (ECM), and (3) physicochemical factors (mechanical stress, hypoxia, and immunosuppressive biomolecules), which collectively drive cancer progression and resistance to therapies [1]. These factors pose significant challenges to effective cancer treatment [2]. The ECM components of the TME can be composed of collagen, elastin, and hyaluronic acid that not only provide structural support but also mediate biological processes through receptors such as integrins, laminin, and CD44, which mediate biological processes [3,4]. Cancer-associated fibroblasts (CAFs) and tumor cells release proteases, including matrix metalloproteinases (MMPs) for extracellular matrix (ECM) remodeling and lysyl oxidase (LOX) for collagen crosslinking. These processes contribute to tissue stiffening, as evidenced in glioblastoma, where increased stiffness impedes drug delivery [5] (Figure 1a). Concurrently, hypoxia resulting from aberrant vasculature enhances glycolytic metabolism, leading to lactate accumulation and acidification of the extracellular milieu (pH 6.5–6.9) [6]. This acidic environment suppresses immune function by impairing the anti-tumor activity of T cells and natural killer (NK) cells, while promoting the infiltration of immunosuppressive M2-type macrophages and regulatory T cells (Tregs) [7,8]. Treg cells are able to inhibit effector T (Te) cells by depleting interleukin-2 (IL-2) and secreting anti-inflammatory cytokines, such as TGF-β/IL-10/IL-35. In addition, myeloid-derived suppressor cells (MDSCs) limit T cell activity through arginase-1 (Arg-1) and inducible nitric oxide synthase (iNOS) [9]. All these complicated TME factors build up physical barriers to resist chemotherapy and low immunoscores that are associated with poor prognosis (Figure 1b). Traditional models struggle to replicate TME’s spatiotemporal complexity for fundamental studies and drug developments, limiting therapeutic discovery. Dynamic hydrogels, with their adaptive properties, offer a promising solution for TME engineering and oncology.

## 2. Dynamic Hydrogels: Designs and Applications

Dynamic hydrogels are three-dimensional networks with dynamic covalent and noncovalent bonds, enabling responsiveness to stimuli like pH, temperature, light, and enzymes [10]. Their properties, including self-healing, shape memory, shear-thinning, and injectability, make them ideal for cancer therapy [11]. By mimicking ECM mechanical and biochemical cues, dynamic hydrogels facilitate precise drug delivery, immune reprogramming, and tumor modeling, reshaping cancer treatment paradigms (Table 1).

### 2.1. Fundamentals of Dynamic Hydrogels for Recreating Tumor Microenvironment and Cancer Therapy

Dynamic hydrogels are ideal carriers for cancer immunotherapy due to their mechanical tunability, rapid gelation, biocompatibility and biodegradability [12,13]. They can serve as drug delivery vehicles, achieving targeted and sustained release by adjusting mechanical properties (e.g., stiffness, crosslinking density), improving efficacy while reducing systemic toxicity [14]. To reconstruct TME for fundamental studies (e.g., drug screening and molecular mechanisms), hydrogels act as scaffolds for 3D tumor models, simulating their microenvironments to study tumor cell behavior and drug response. For instance, by tuning chemical composition and physical properties (e.g., pore size and stiffness), hydrogels can replicate TME conditions, such as the stiffness of breast cancer models (0.5–50 kPa in vitro), enabling studies of tumor cell migration and drug response [15,16].

In immunotherapy, hydrogels enhance the delivery of immune checkpoint inhibitors, such as anti-programmed death-1 (anti-PD-1) antibodies, improving therapeutic outcomes in cancers like non-small-cell lung cancer and hepatocellular carcinoma [17,18,19]. For example, pH-responsive hydrogels loaded with anti-PD-1 antibodies have shown enhanced T cell infiltration and reduced tumor growth in preclinical models [20]. Dynamic hydrogels are also being explored for chemotherapy, radiotherapy, thermotherapy, and photodynamic therapy, though many applications remain in preclinical stages [21,22,23]. Recent developments include fibronectin-based hydrogels for cell adhesion, antibody-loaded nanoparticle-dispersed hydrogels for targeted drug delivery, and in situ formed βCD-modified alginate (ALG-βCD) hydrogels that recruit CCR9+ CD8+ T cells via chemokine CCL25, achieving up to 87.71% anticancer activity in mouse models [24]. These advancements highlight the potential of dynamic hydrogels to modulate the TME and enhance immune responses, paving the way for innovative cancer therapies.

### 2.2. Design and Classification of Dynamic Hydrogels

The design of dynamic hydrogels is categorized based on material source and reaction mechanism to address specific TME challenges. Dynamic cross-linking, a key property, enables self-healing and injectability through reversible bond breaking and reorganization, adapting to the TME’s dynamic conditions. For instance, Schiff base bonding is based on the reversible condensation reaction between amino and aldehyde groups and is pH responsive. Carboxymethyl chitosan and oxidized cellulose nanocrystals crosslinked via Schiff base bonds can be solubilized on-demand by amino acid competitive bonding, showcasing controlled degradation potential [25]. Host–guest interaction, such as β-cyclodextrin (β-CD) and adamantane (Ad), offers a high binding constant and rapid self-healing, though they require a precise molecular ratio, complicating the fabrication [26]. We have summarized the representative types of dynamic hydrogels and their biomedical applications in Table 2.

#### 2.2.1. Natural vs. Synthetic Hydrogels

Natural and synthetic hydrogels offer distinct advantages and limitations, driving the development of multicomponent systems. For natural hydrogels, hyaluronic acid (HA) promotes cell adhesion, regulates embryonic development, and enhances ECM secretion, but degrades rapidly and has low mechanical strength. Recent HA-based hydrogels incorporate MMP-sensitive peptides, extending degradation time and improving tumor modeling [27]. In addition, chitosan–hyaluronic acid composite hydrogels can be used for wound repair and to accelerate angiogenesis [28]. Similarly, collagen gels (such as rat tail collagen) can directly mimic the extracellular matrix (ECM), promoting the differentiation of dental pulp stem cells into odontoblasts, thereby significantly enhancing vascularization and tissue formation in pulp regeneration [29]. Fibrin gel derived from coagulation factors is used for spinal cord injury repair. Peng et al.’s research indicates that implanting fibrin gel loaded with nerve growth factor into rat spinal cord injuries promotes axonal regeneration and reduces glial scar formation [30].

Natural hydrogels exhibit low mechanical strength, typically ranging from 1 to 100 kPa. The elastic modulus can be adjusted by varying crosslink density, while their long-term mechanical stability is rapidly weakened due to enzymatic/hydrolytic degradation. For example, collagen-based degradation cycles span approximately 7–21 days, aligning with tissue regeneration rates [31]. Although natural hydrogels offer high biocompatibility, they often show low drug loading capacity (generally 5–12% *w*/*w*) [32]. For instance, the chitosan-gelatin hydrogel used for the drug 5-Fluorouracil (5-FU) exhibits a drug loading efficiency of 7.8 ± 0.4% (*w*/*w*), constrained by their small mesh size [33].

Synthetic Hydrogels such as polyethylene glycol (PEG) hydrogels offer high controllability, enabling patterned surface modifications (e.g., laser photopolymerization) to load vascular endothelial growth factor (VEGF) for directed vascularization [34]. Functionalization is required for biocompatibility, as seen in PEG hydrogels modified with RGD peptides for cell adhesion [35]. In the nerve regeneration scaffold, NGF and BDNF growth factors are layered and fixed in different regions of the PLGA hydrogel to guide axonal growth [36]. Another synthetic hydrogel, polyacrylamide (PAAm), exhibits high mechanical strength (elastic modulus ≥ 10 kPa) to mimic bone tissue hardness, promoting integrin clustering and activating the FAK signaling hub in MSCs. This drives the RhoA/ROCK pathway (enhancing cytoskeletal tension) and ERK/MAPK pathways, ultimately upregulating core transcription factors Runx2 and Osterix. This initiates the osteogenic gene expression program, enabling differentiation into osteoblasts [37,38]. Temperature/pH dual-responsive hydrogels (such as PNIPAAm-PAA) are used for targeted drug delivery to tumors. For example, oral insulin hydrogels swell and release drugs at intestinal pH while remaining stable in gastric fluid [39,40].

The mechanical properties of synthetic hydrogels can be extensively tuned, with stiffness ranging from 0.1 kPa to 10 MPa. The toughness can be significantly enhanced through dual-network designs (e.g., polyelectrolyte/polyacrylamide composites) or chemical crosslinking reinforcement. For instance, hydrogels incorporating “sacrificial weak bonds” achieve compressive moduli in the MPa range and exhibit fracture energy increases exceeding tenfold [41,42]. Second, these hydrogels can achieve drug loading capacities of 20–40 wt%, such as polyacrylic acid hydrogels loaded with charged drugs via ion exchange, which shows a 30–50% efficiency increase [42,43]. Additionally, they exhibit extended degradation cycles (weeks to months). Chemically crosslinked types (e.g., ester bonds) require 30–60 days for hydrolysis, while reversible crosslinks (e.g., dynamic covalent bonds) reduce the degradation duration to 1–4 weeks [42].

Combining natural and synthetic components enhances functionality. Alginate-β-cyclodextrin (ALG-βCD hydrogels) use beta-cyclodextrin (β-CD) to capture adamantane-modified anti-PD-1 antibodies (Ad-aPD1) via host–guest interactions, while immobilizing chemokine CCL25 to recruit CCR9+ CD8+ T cells. This system reduced tumor volume by 70% in mouse models by blocking the PD-1/PD-L1 pathway and reversing T cell exhaustion. Photocrosslinked networks, combining Schiff base bonds for self-healing with UV-triggered acrylate polymerization, enhance mechanical toughness, achieving compression moduli up to 4.33 kPa [44]. These networks improve fracture energy, making them suitable for injectable applications.

#### 2.2.2. Multi-Stimuli Responsive Hydrogel

Multi-stimuli responsive hydrogels integrate responses to pH, reactive oxygen species (ROS), temperature, and enzymes, enabling precise drug release tailored to the TME’s physicochemical properties [1]. These hydrogels leverage the TME’s acidic extracellular pH (6.5–6.9), elevated intracellular GSH (2–10 mmol/L vs. 0.1–1 mmol/L in normal cells), high ROS levels, and overexpressed enzymes like matrix metalloproteinases (MMPs).

For pH-responsive hydrogels, they exploit ionizable groups (e.g., carboxyl groups, amino groups) on polymer chains. Anionic hydrogels shrink at low pH due to protonated carboxyl groups, reducing electrostatic repulsion and triggering drug release, whereas cationic hydrogels swell due to protonated amino groups, increasing hydrophilicity [45,46]. For example, cisplatin-containing polyethylene glycol-poly(lactic acid) (PEG-PLA) hydrogels release platinum nanoparticles in the acidic TME (pH 5.5–6.8), and with secondary GSH-triggered release, improving efficacy and reducing systemic toxicity [47].

For enzyme-sensitive hydrogels, matrix metalloproteinases (MMPs)-sensitive peptide sequence (e.g., GPLGVRG) can be utilized as a crosslinker of the hydrogel, which can be specifically degraded by the MMPs (e.g., MMP-2 or -9) overexpressed in tumor tissues to release the encapsulated drugs. For example, PEG-vinylsulfone copolymerized with MMP-recognizing peptides forms hydrogels that are cleaved by MMP at the tumor site to release drugs or recruit immune cells [48]. Recent advancements include MMP-sensitive hydrogels releasing IL-12, enhancing T cell activation in preclinical models [49].

Temperature-sensitive hydrogels have made a remarkable contribution to localized therapy at body temperature (37 °C). For example, Pluronic F127 hydrogel gelled at body temperature, loaded with triptolide (TPL) achieved a 14-day sustained release, reducing breast tumor growth by 60% in mouse models [50].

ROS-sensitive hydrogels, such as those with thioketal linkages, degrade in high ROS environments (e.g., 50–100 μM in tumors vs. 20 μM in normal tissues) [51,52], releasing drugs like pazopanib to inhibit metastasis [53]. Recent studies combine ROS and pH responsiveness, achieving sequential drug release for enhanced tumor ablation [54]. In addition, a new type of multifunctional cerium-based nanocomposite hydrogel can be used for skin wound healing and regeneration. It is temperature-sensitive, injectable, and self-healing, and can effectively remove reactive oxygen species [55].

The hydrogels mentioned above exhibit varying degrees of sensitivity in their stimulus responses. For temperature/pH-responsive types, phase transition times occur within minutes. Temperature-responsive hydrogels typically exhibit a low critical solution temperature (LCST) range of 30–40 °C, while pH-responsive types swell or shrink within the pH range of 4–8 [56]. For light-responsive hydrogels, gel-to-sol transitions occur within seconds to minutes under UV/visible light irradiation, with a light intensity threshold of approximately 10mW/cm^2^ [57]. In multi-stimulus-responsive hydrogels, combined stimuli (e.g., light + pH) accelerate responses, enhancing sensitivity by 2–5 times. However, microstructural heterogeneity may cause localized response delays [56,58]. Additionally, the stiffness of these multi-stimulus-responsive hydrogels can be dynamically tunable. For instance, the storage modulus (G’) of light-responsive hydrogels decreases by 50–90% (e.g., from 10 kPa to 1 kPa) after irradiation [41]. Reduction-responsive hydrogels exhibit a 30–70% reduction in G’ in glutathione environments [41]. Apart from their controllable mechanical properties, the drug-controlled release rate can also be fine-tuned and robust. pH/temperature dual-responsive hydrogels can achieve 90% drug release in the tumor microenvironment (pH 6.5, 40 °C) but <10% release under physiological conditions [42,43]. Also, enzyme-responsive hydrogel (e.g., Gelatin-HA hydrogel) can achieve stepwise degradation through a combination of ester bond hydrolysis (>14 days) and enzymatic cleavage (<7 days) [41,42]. This demonstrates the broad application prospects of multi-stimulus-responsive hydrogels in the pharmaceutical field.

**Table 1:** Quantitative Comparison Table of Key Hydrogel Properties.

Performance Parameters	Natural Hydrogels	Synthetic Hydrogels	Multi-Stimuli Responsive Hydrogel	Reference
Mechanical stiffness range	0.1–10 kPa (collagen); 0.1–1.5 kPa (agarose)	0.1–150 kPa (PVA); 0.01–7360 kPa (PEG)	Dynamically Adjustable: Light-responsive (10 kPa → 2 kPa); pH-responsive (modulus change ± 50%)	[38]
Stimulus-response sensitivity	No inherent response	Requires functional modification	pH response: Swelling ratio 2.98–10, response time 60–300 s (PAAc); Temperature response: Swelling ratio 2.98–20, response time 300–540 s (PNIPAAm); Light response: Contraction rate 15%, response time < 120 s	[59]
Drug loading efficiency (EE%)	65–90% (limited by low mechanical strength)	70–98% (capable of designing high drug-loading structures)	Smart Controlled Release: pH-triggered release rate > 80%; Temperature-triggered sustained release cycle: 1–7 days	[60]
degradation cycle	Days–weeks (enzymatic degradation dominant)	Weeks–months (hydrolysis controllable)	Stimulus-dependent degradation: Photodegradation (hour-scale); Enzyme/pH synergistic degradation (day-scale)	[61]
Biocompatibility	High (low immunogenicity)	Moderate–High (potential residual toxic crosslinking agents)	Moderate (requires safety verification)	[62]

#### 2.2.3. Multi-Drug Loading Hydrogels

Multifunctional loading is critical for the role in the TME, enabling co-delivery of drugs, cytokines, and oxygen carriers that need to be integrated in order to target a variety of tumor tissues and organ pathways. For instance, nanostructured lipid carriers (NLCs) combined with chitosan-triphosphate hydrogels enhance paclitaxel encapsulation up to 85%, improving drug delivery efficiency [42]. In addition, IL-12-loaded hydrogels can activate T cells and reverse the immunosuppressive microenvironment of bone metastases [63]. Similarly, combining PD-1 antibodies with TGF-β inhibitors can synergistically inhibit Treg cells and enhance CD8+ T cell infiltration [64]. Moreover, oxygen carriers such as hemoglobin or perfluorocarbons (PFCs) can alleviate tumor hypoxia and enhance the effect of photodynamic therapy (PDT) [65], as seen in Bionic polydopamine hydrogels with antimicrobial and pro-angiogenic effects [66].

Recent advancements include smart hydrogels responding to multiple stimuli (e.g., pH, ROS, temperature, glucose), enabling precise drug release control. Peptide-based supramolecular hydrogels conjugate drugs to peptides, forming amphiphilic conjugates that enhance solubility and targeting, such as RGD-incorporated pentapeptide hydrogels targeting integrins. Cyclodextrin-based hydrogels encapsulate hydrophobic drugs, offering multi-drug delivery and pH-responsive release, while nanoparticle-integrated hydrogels enhance cellular uptake and minimize systemic toxicity. Sequential drug delivery hydrogels release multiple drugs in phases, optimizing effectiveness for complex tumors.

#### 2.2.4. Immune-Activated Hydrogels

Immune-activated hydrogels modulate the TME by controlling drug release, recruiting immune cells, and enhancing immune responses [67]. They address TME challenges such as hypoxia and immunosuppression by delivering checkpoint inhibitors (e.g., anti-PD-1) or carbonic anhydrase IX (CA IX) blockers, relieving hypoxia and activating CD8+ T cells. Secondly, Self-assembled short peptide (PepABS-py) hydrogels composed of self-assembled peptide nanofibers adsorb immunosuppressive exosomes, reducing their diffusion. When coupled with PD-L1 antibodies, these hydrogels neutralize exosomal immunosuppressive signals, enhancing anti-tumor immunity in preclinical studies [68]. Recent advancements include hydrogels co-delivering IL-15 and anti-PD-1, significantly improving the complete remission rate in pancreatic cancer models by boosting T cell infiltration [69].

**Table 2:** Summary of Dynamic Hydrogel Types and Applications.

Hydrogel Type	Stimulus/Mechanism	Application	Reference
MMP-Sensitive Hydrogel	Enzyme (MMP-2/9)	Drug release, immune recruitment	[1]
pH/GSH Hydrogel	pH, GSH	Drug delivery (adriamycin)	[20]
ALG-βCD Hydrogel	Host-guest, chemokine	T cell recruitment	[24]
Photocrosslinked Network	Schiff base, UV	Mechanical toughness	[25]
Pluronic F127	Temperature	Localized therapy (breast cancer)	[50]
CS15-FTP-gel	hypoxia induction	maximize chemotherapeutic efficacy (AQ4 toxicity)	[53]
cerium-based nanocomposite hydrogel	Temperature/remove ROS	Skin wound healing and regeneration	[55]
PepABS-py	Exosome adsorption	Immunotherapy (PD-L1 neutralization)	[68]
Photoresponsive hydrogel	Specific wavelength illumination (UV/Vis/NIR)	soft robotics	[70]

## 3. Multifunctional Applications in TME Engineering and Therapy

Dynamic hydrogels leverage nanotechnology and TME-specific cues for precision drug delivery, 3D tumor modeling, combination therapy, and immune reprogramming, addressing the complexity of the TME (Table 3).

### 3.1. Precise Drug Delivery

pH/GSH dual-responsive hydrogels utilize the extracellular pH value (6.5–6.9) and intracellular glutathione (GSH) value (2–10 mmol/L) of the tumor microenvironment (TME) to selectively release drugs at tumor sites [71]. Doxorubicin–peptide composites can self-assemble into nanoscale hydrogels, releasing doxorubicin through the hydrolysis of acid-sensitive amide bonds, thereby reducing toxicity and enhancing membrane permeability [53]. Liposome-peptide nanoparticles can prolong drug residence time, improving efficacy by 65% in a breast cancer model [72]. pH-responsive nanoplatforms, such as hollow mesoporous silica nanoparticles loaded with polyaniline and indocyanine green, can activate photothermal/photodynamic synergistic effects in acidic TME, achieving 90% tumor ablation efficiency [72]. Additionally, precise targeting of tumor cells can be achieved by coupling peptides with specific targeting molecules (e.g., tumor-associated receptors), such as the D-Lys^6^-GnRH peptide carrier conjugated to 2-pyridine doxorubicin (AN-201) via ε-amino covalent bonding, targeting gonadotropin-releasing hormone (GnRH/LHRH) receptors. A single injection of 250 nmol/kg AN-207 resulted in complete regression of MX-1 breast cancer mouse tumors (100% cure rate) with no significant toxicity [73]; by conjugating the somatostatin analog RC-121 with AN-201, it primarily targets somatostatin receptor subtypes SSTR2 and SSTR5. In NCI-N87 gastric cancer nude mice, AN-238 reduced tumor volume by 50% (*p* < 0.05), significantly outperforming the control group [74]. The photothermal properties activated by plasma promote tumor tissue differentiation and targeted damage. Activating photothermal/photodynamic synergy in an acidic tumor microenvironment (TME) significantly enhances tumor ablation efficiency [75]. For example, at 53 °C, reactive oxygen species (ROS) production reaches the cytotoxic threshold (>100 μM), leading to lipid peroxidation and DNA fragmentation. (Gold nanorods induced a threefold increase in ROS production under 808 nm laser irradiation, synergistically reducing 4T1 cell survival to below 15% with photothermal effects [76]); AuNR/GO dual-plasmonic nanohybrid materials combined with chemotherapy in an acidic TME achieved an ablation rate of 89%, with a 50% reduction in HSP70 expression, indicating more thorough cell killing [77].

**Table 3:** Application of pH/GSH-responsive hydrogels in precise drug delivery.

Technology	Hydrogel Type	Application	Outcome	Reference
pH/GSH dual response release	Adriamycin-peptide nanogel	Breast cancer treatment	Reduced toxicity, enhanced membrane permeability, prolonged drug retention time, and 65% improvement in efficacy.	[72]
Photothermal/photodynamic synergy	Polyaniline-indocyanine green hydrogel	tumor ablation	Tumor ablation efficiency of 90% in acidic TME.	[72]
Targeted molecular coupling	D-Lys^6^-GnRH peptide hydrogel	Precise targeting of tumor cells	A single injection of 250 nmol/kg AN-207 resulted in 100% tumor regression in MX-1 breast cancer mice.	[73]
Somatostatin receptor targeting	RC-121 coupled with AN-201 hydrogel	Precise targeting of tumor cells	AN-238 reduced tumor volume by 50% in NCI-N87 gastric cancer nude mice (*p* < 0.05).	[74]
Gold nanorods enhance ROS	AuNR/GO hybrid hydrogel	tumor ablation	ROS production increased threefold, with a survival rate of <15% for 4T1 cells.	[24,76]

### 3.2. Bioprinting Technology for Tumor Modeling

Bioprinting technology utilizes computer-aided design (CAD) to create complex TME models [78] and employs bioinks such as GelMA hydrogels to simulate the stiffness of the ECM (0.5–50 kPa in vitro, 94–147 kPa in vivo for breast cancer) [79]. The adjustable properties of GelMA (e.g., 5–20% *w*/*v* concentration, 50–90% substitution rate) enable precise replication of tumor-specific microenvironments, thereby facilitating angiogenesis and metastasis research [80]. For example, 3D-printed GelMA scaffolds seeded with patient-derived breast cancer organoids achieved an 85% accuracy rate in predicting chemotherapy response, compared to only 50% for 2D models [81]. Recent advancements involve incorporating immune cells (such as macrophages and T cells) into bioinks, enabling co-culture models to simulate immune-tumor interactions with 70% fidelity to in vivo conditions [82]. These models support personalized drug screening, reducing clinical trial failure rates by 30%. This approach provides new tools for personalized therapy and drug screening.

**Figure 2:**
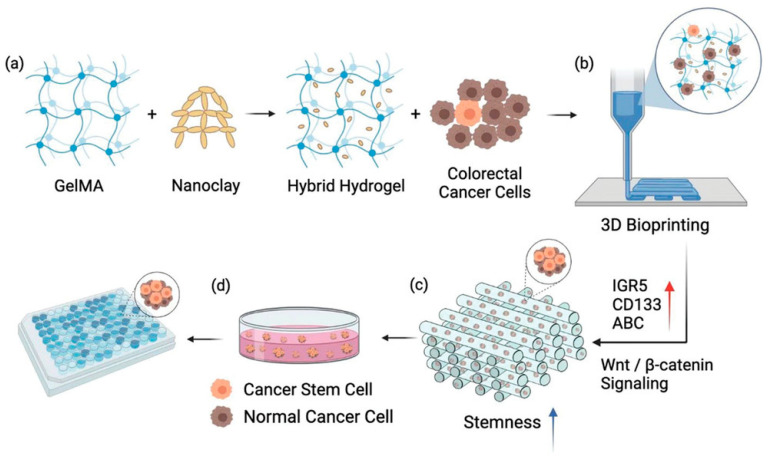
Schematic of using 3D bioprinted GelMA-nanoclay hydrogel to construct CSC-enriched spheroid models for drug screening. (**a**) GelMA-nanoclay hydrogel preparation. (**b**) 3D bioprinting process with cell-laden GelMA-nanoclay hydrogel. (**c**) Mechanism of CSC enrichment in GelMA-nanoclay hydrogels. (**d**) Spheroids isolated from hydrogels followed by CSC targeting drug screening. Figure is adapted from [83].

Furthermore, studies have found that incorporating nanoclay into GelMA hydrogels promotes the formation of colorectal cancer (CRC) cell spheroids, suggesting that GelMA–nanoclay hydrogels may possess the ability to induce and enrich cancer stem cells (CSCs). Therefore, a CSC-enriched spheroid model for drug screening was constructed using 3D bioprinted GelMA–nanoclay hydrogels (Figure 2). Through in vitro and in vivo functional assays, it was demonstrated that the spheroids derived from the 3D-printed GelMA–nanoclay hydrogels exhibited elevated stemness due to the activation of Wnt/β-catenin signaling. Compared to traditional CSC enrichment models, the CSCs derived from GelMA–nanoclay hydrogels not only showed the expected enhanced stemness but, more importantly, exhibited higher consistency, yield, and increased sensitivity to anti-CSC compound treatments. This provides a new strategy for the design and preparation of advanced CSC enrichment models, with potential applications in high-throughput screening of anti-CSC compounds [83].

3D bioprinting has been utilized in tissue engineering to complex in vitro models of tissues such as liver, heart, and skin, enabling advanced drug screening, disease modeling, and potential organ fabrication [84]. For example, 3D-bioprinted liver tissues can maintain metabolic functions for several weeks to several months, or for up to five months [85]. Animal-component-free 3D bioprinted liver models achieve viability rates as high as 97–101% within 15 days and are capable of simulating drug toxicity testing [86], showing strong correlations with in vivo pharmacokinetics in some studies. Additionally, in tumor research, 3D bioprinting facilitates the creation of patient-specific cancer models for personalized drug delivery systems, with reports that the encapsulation efficiency of water-soluble drugs in superhydrophobic matrices exceeds 95% [87]. However, regarding the specific 3D-printed GelMA hydrogel scaffolds cited for 85% accuracy in chemotherapy response prediction, the current literature does not provide details on clinical sample verification, such as matching with patient-derived organoids or human trials [88]. Research remains primarily in preclinical stages, focused on in vitro and animal models, with no documented small-scale clinical validations to date.

Advances in 3D bioprinting also offer a promising solution to traditional obstacles (such as forming stable and perfusable blood vessels in vitro), as it enables precise control over the spatial arrangement of cells, biomaterials, and signaling molecules. By leveraging 3D bioprinting, researchers can now create complex in vitro models that accurately replicate the 3D structure of the tumor microenvironment. By carefully coordinating the deposition of cells, biomaterials, and growth factors, complex vascular networks can be created within these models, which closely resemble the complexity observed in vivo. This enables a thorough exploration of the mechanisms underlying cancer angiogenesis, thereby providing important insights into the mechanisms of tumor angiogenesis and metastasis [89].

### 3.3. Microfluidics-Hydrogel Coupling

Microfluidic technology provides key advantages in cell culture, including low shear stress (typically ≤ 1 mPa), high throughput (processing up to 10^4^–10^6^ cells per device), and precise environmental control, making it ideal for studies on cell differentiation, behavior, and single-cell analysis [90]. It supports applications in disease diagnosis, such as detecting circulating tumor cells (CTCs) with sensitivities reaching 90–95% for early cancer prognosis, as well as exosome and cell-free DNA analysis [91]. In oncology, microfluidic chips simulate the tumor microenvironment for drug screening, with models achieving up to 85–95% predictive accuracy for treatment responses in colorectal cancer (CRC) simulations. For example, integrated microfluidic-hydrogel systems have been used to culture patient-derived CRC organoids, enabling high-throughput drug testing with success rates for organoid establishment ranging from 50 to 90%, significantly outperforming patient-derived xenografts (PDXs), which have only 10–30% success rates. These models also allow for co-culture with immune cells, replicating aspects of the tumor microenvironment and showing comparable drug response profiles to in vivo studies in some cases. Compared to traditional biopsy methods, which provide static snapshots with limited predictive power (e.g., 60–70% concordance with clinical outcomes), microfluidic-organoid platforms offer dynamic, real-time monitoring and higher fidelity to patient heterogeneity, though they lack full vascular and immune integration [92]. Regarding the specific 90% accurate colorectal cancer organoid model using microfluidic-hydrogel coupling, no small-scale clinical trial data or direct comparisons with biopsy-based methods are detailed in the existing literature. Progress is mostly confined to preclinical and in vitro validations, with ongoing efforts to bridge toward clinical translation through patient-derived systems [93].

Microfluidics technology can simulate the construction of three-dimensional vascular networks within the body, surpassing two-dimensional models in terms of vascularization simulation. Microfluidic chips based on hydrogels utilize alginate-gelatin dual-network hydrogels, which form hollow tube structures through gel microfabrication, supporting endothelial cell adhesion and vascularization [53]. Secondary cross-linking enhances the chip’s durability, generating multi-directional fluid shear forces (e.g., 5–15 dynes/cm^2^ [94]), promoting endothelial cell adhesion, and achieving 75% vascular simulation efficacy compared to natural veins and arteries [94]. For example, a research group at Harvard Medical School developed coaxial bio-printed alginate-gelatin hydrogels, forming functionalized single-layer and double-layer vessels with structural similarity to human vessels up to 80% [94].

Microfluidic-hydrogel systems enable dynamic studies of substance diffusion and angiogenesis, supporting long-term incubation (up to 2 months) for real-time monitoring of thrombus formation and endothelial barrier function recovery [75]. Recent innovations include patient-specific organoid integration, achieving up to 90% accuracy in establishing metastatic invasion models on microfluidic chips seeded with colorectal cancer organoids [95]. These systems also facilitate immune cell perfusion, simulating TME immune suppression, and improving immunotherapy screening by 65% (Table 4).

### 3.4. Combined Therapy

Combined therapy integrates chemotherapy, radiotherapy, immunotherapy, and emerging modalities (such as photothermal therapy and catalytic therapy) to achieve synergistic effects and overcome the limitations of single therapies. For example, the combination of chemotherapy and immunotherapy can enhance the antitumor activity of immunotherapy, improving overall survival; radiotherapy combined with immunotherapy can improve the tumor microenvironment (TME) [96]; MoS_2_-DOX nanocomposites exhibit an inhibition rate of up to 92.09% against liver cancer cells under near-infrared light, significantly outperforming monotherapy [97]; photothermal therapy can also enhance immunogenicity by releasing tumor antigens, and when combined with immune checkpoint inhibitors (such as PD-1/PD-L1 inhibitors), it can enhance systemic antitumor immune responses [96]. Dynamic hydrogels are multifunctional platforms that respond to tumor tissue-mediated factors (such as acidic pH and high ROS) and deliver multiple therapeutic drugs. Compared to monotherapy, dynamic hydrogel systems achieve inhibition rates of 70–90% in specific tumor models. For example, MoS_2_ nanocomposites loaded with DOX achieved an inhibition rate of 92.09% against liver cancer cells [97].

Chen Xin’s team developed a micro-nano platform combining cysteine-modified gold nanoparticles with amino-functionalized mesoporous silica nanoparticles, using ferrous ion bonding to block drug-loaded mesopores. In an acidic mesoporous medium (pH 5.5–6.8), the gold nanoparticles detach, releasing chemotherapy drugs (such as doxorubicin), while self-assembling for near-infrared photothermal therapy (PTT). Ferrous ions catalyze the production of hydroxyl radicals from cytoplasmic hydrogen peroxide (100–1000 μM in tumors), thereby enhancing oxidative damage. This triple therapy reduced tumor size in melanoma and breast cancer mouse models and improved survival rates compared to chemotherapy alone [98]. Recent advances include the incorporation of pH/ROS-responsive hydrogels to encapsulate the nanoplatform. pH/ROS dual-responsive hydrogels achieve a drug release rate of up to 95% within 24 h in an inflammatory microenvironment (pH ≤ 6.8, ROS ≥ 50 μM), whereas traditional PLGA microspheres release only 65%. By reducing premature release, targeted delivery is achieved, increasing drug retention by approximately 30% [99].

Alterations in lipid metabolism in the tumor microenvironment (TME) produce immunosuppressive metabolic products that inhibit the function of effector T cells. Inhibition of inositol-requiring enzyme 1 (IRE1α) can reprogram T cell lipid metabolism, prevent aging, and enhance antitumor immunity. For example, the IRE1α-XBP1 pathway inhibits TAGLN2 expression, leading to impaired lipid metabolism in CD8+ T cells; overexpression of TAGLN2 enhances T cell antitumor activity [100]. Peng Yiman’s team developed a weakly pathogenic Salmonella strain capable of secreting flagellar protein B (FlaB) from Vibrio vulnificus, and observed significant antitumor effects in various mouse tumor models. Additionally, the study found that FlaB-mediated tumor suppression is closely associated with the TLR4 signaling pathway, while TLR5 signaling further enhances the host’s immune response. Encapsulating these formulations in hydrogels enhances local delivery and improves efficacy [101].

Oxygen-sensitive hydrogels trigger the cleavage of ketone–thioether bonds in tumor regions with high ROS expression (e.g., H_2_O_2_, at concentrations up to 100–1000 μM), releasing pazopanib. By inhibiting vascular endothelial growth factor receptors (VEGFR), tumor angiogenesis is blocked, metastasis is inhibited, and hypoxia is alleviated [53] (solid tumors, due to vascular malformations and high metabolic activity, form hypoxic cores with oxygen levels typically below 0.5%, with overall ranges of 1–5%, while normal tissues have oxygen levels of 5–15% [102]). This increases oxygen saturation in the tumor microenvironment (TME), enhancing chemotherapy efficacy by 40–60% in lung cancer models [103]. A patent from Anhui University of Technology shows that a ROS-responsive chitosan gel prodrug system can increase the killing efficiency of cisplatin on cancer cells by approximately 50% while reducing toxicity to normal cells by 70%. In lung cancer models, cisplatin therapy combined with hydrogels is expected to increase metastasis inhibition rates from 30 to 40% with traditional chemotherapy to 70–80% (based on inferences from studies with similar mechanisms) [104].

Emerging hybrid systems integrate nanoparticles (e.g., gold nanorods) with multi-stimulus hydrogels (pH/ROS/temperature-responsive) for continuous therapy. For example, PTT mediated by nanoparticles (e.g., iron oxide nanoparticles) destroys tumor-associated regulatory T cells (Tregs), and when combined with immune checkpoint blockade (ICB) therapy (e.g., anti-PD-1 antibodies), significantly enhances tumor ablation efficacy. By combining PTT, chemotherapy, and immunotherapy, a complete tumor regression rate of 55.5% was achieved in a glioblastoma model. Additionally, studies showed that PTT significantly reduced the proportion of Tregs in the tumor microenvironment (from 42.2% to 11.9%), further enhancing the efficacy of immunotherapy [105].

### 3.5. Immune Reprogramming

Immune reprogramming utilizes hydrogels to deliver immunomodulatory agents, promoting dendritic cell (DC) maturation, T cell activation, and tumor clearance. Dynamic hydrogels can create a local inflammatory microenvironment to counteract TME immune suppression (such as PD-L1 overexpression and Treg dominance), thereby enhancing antitumor immunity by 65–85% [106].

**Figure 3:**
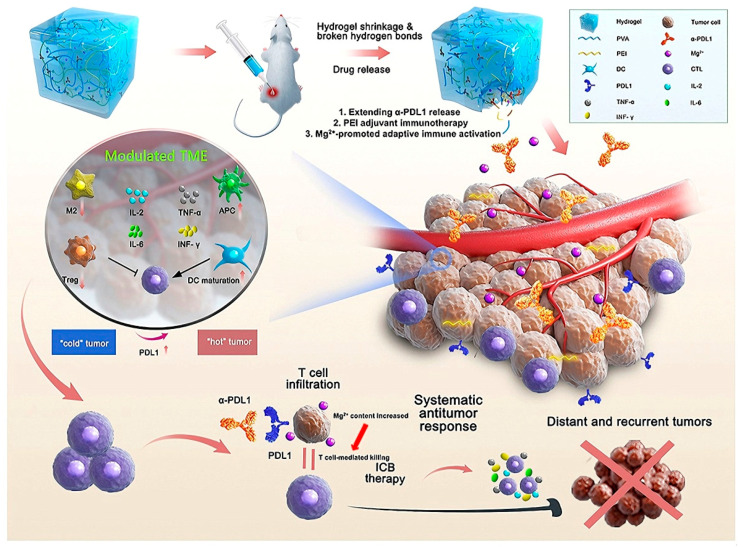
Schematic illustration of an anti-PD-L1 antibody-loaded PVA-PEI hydrogel (α-PDL1/PEIGel) for boosting and enhancing immune checkpoint blockade (ICB) therapy via local administration. PEIGel exerts innate modulatory effects on the tumor microenvironment, leading to adjuvant effects that reverse the immunologically “cold” phenotype to a “hot” phenotype by upregulating PDL1 expression and promoting M1-like macrophage polarization. Figure is adapted from [108].

Toll-like receptor (TLR) 7 or 8 agonists conjugated with peptide-DNA hydrogels can reprogram tumor-associated macrophages (TAMs) from an M2 (pro-tumor) phenotype to an M1 (anti-tumor) phenotype, thereby improving DC maturation and antigen presentation capacity. This induces the expansion of anti-tumor T cells, significantly inhibiting tumor growth in melanoma models [107]. Hydrogels can mimic an immunogenic microenvironment, increasing DC activation by 70% compared to systemic TLR delivery [71]. Recent advances include STING agonist-loaded hydrogels, which promote type I interferon production and achieve an 85% tumor regression rate in pancreatic cancer models [107]. The PVA-PEI hydrogel (α-PDL1/PEIGel) loaded with anti-PD-L1 antibodies promotes and enhances immune checkpoint blockade (ICB) therapy through local administration. The PEIGel encapsulates the anti-PDL1 antibody, which works synergistically to eliminate primary tumors and distant metastases, and prevent tumor recurrence after surgical resection (Figure 3) [108].

CAR-T cells directly kill tumor cells by targeting specific antigens on the surface of tumor cells. Hydrogels loaded with CAR-T cells provide a local inflammatory microenvironment that promotes CAR-T cell proliferation and activation. For example, hyaluronic acid hydrogels can sustain IL-15 release, enhancing CAR-T cell energy and killing capacity. Hydrogels can also prevent cytokine diffusion through physical barriers while allowing CAR-T cells to actively migrate to tumor sites for precise killing. Additionally, hydrogels can be combined with other therapies, such as anti-PD-1 antibodies, to further block the PD-1/PD-L1 signaling axis and enhance CAR-T cell function [109].

Hydrogels loaded with TLR agonists primarily promote antigen presentation and immune activation by activating dendritic cells, while hydrogels loaded with CAR-T cells enhance CAR-T cell proliferation, activation, and killing capacity by providing a local inflammatory microenvironment and physical barrier. Both effectively promote tumor clearance but operate through distinct mechanisms.

## 4. Challenges to Clinical Translation

Translating dynamic hydrogels from preclinical research to clinical applications faces significant hurdles, including biocompatibility, scale-up, regulatory barriers, and model limitations. These challenges impede the adoption of hydrogels for TME engineering, despite their promise in drug delivery, 3D modeling, and immunotherapy.

### 4.1. Biocompatibility

Residual crosslinking agents and shear-induced cytotoxicity can lead to biocompatibility issues, affecting the safety and usability of hydrogels. Glutaraldehyde is a common crosslinking agent that covalently binds to the amino groups and cysteine sulfhydryl groups of proteins, disrupting the integrity of cell membranes and enzyme activity. In vivo, glutaraldehyde is metabolized via β-oxidation into carbon dioxide, but high concentrations (e.g., >0.1% *w*/*v*) can lead to intermediate accumulation, causing mitochondrial dysfunction, tissue fibrosis, and organ toxicity in 30–50% of preclinical models [110]. Alternatives such as genipin or enzyme crosslinkers (e.g., transglutaminase) show significantly low cytotoxicity [111], but their scalability is limited.

During injection or processing, fluid shear stress (5–20 dyn/cm^2^) activates mechanosensitive receptors (e.g., NKG2D), triggering PI3K and MAPK pathways, granzyme release, and ROS generation. Immune cells (e.g., NK cells, T cells [112]). The synergistic effect of NK cells with fluid shear stress results in significantly higher CTC lysis rates compared to NK cells alone or FSS treatment, indicating that shear stress significantly enhances killing efficacy [113]. Shear stress can also enhance NK cell cytotoxicity against circulating tumor cells by 50%, producing a dual effect that complicates treatment design [114]. New strategies, such as shear-thinning hydrogels, can reduce cytotoxicity by 70%.

### 4.2. Scaling Up

Scaling up dynamic hydrogel production to meet Good Manufacturing Practice (GMP) standards poses challenges in terms of reproducibility due to inconsistent raw material quality, non-standardized equipment, inconsistent environmental control, insufficient personnel training, and material instability. For example, the grafting rate and concentration fluctuations of GelMA directly affect the Young’s modulus of the hydrogel. Experimental data show that the Young’s modulus of GelMA at 15% concentration is 98.43 ± 7.71 kPa (approximately 7.8% deviation), while the deviation at 6% concentration is 1.62 kPa (approximately 11.1%), indicating that hardness deviations between batches can reach 10–20% [114]. Small and micro-sized industrial enterprises suffer from accelerated equipment wear, increased material waste, high employee turnover rates (10% per month), and skill shortages, all of which directly hinder production progress [115]. Strict GMP procedures (including automated bioreactors and real-time quality monitoring) can reduce scrap rates by 50% [116], but high capital expenditures and cleanroom construction costs (US$7000 per square meter) pose significant cost barriers [117].

The synthesis parameters of GelMA, such as gelatin concentration, reaction temperature, pH, and reaction time, significantly influence the crosslinking density and mechanical properties of GelMA.

By optimizing these parameters, precise control over the hardness of GelMA can be achieved. For instance, studies have shown that by adjusting the gelatin concentration (10–20 *w*/*v* %) and reaction temperature (35–50 °C), GelMA hydrogels with high crosslinking density can be prepared. Furthermore, by optimizing the synthesis conditions, batch-to-batch variations can be reduced, improving production consistency [118]. Research has indicated that the incorporation of graphene oxide is positively correlated with the hardness of GelMA. The hardness and mechanical properties of GelMA can be enhanced by introducing composite materials such as graphene oxide. Moreover, standardizing the synthesis process (e.g., reaction time, temperature, light exposure conditions) can improve the batch consistency of GelMA. For example, performing photocrosslinking under constant temperature and illumination conditions can reduce hardness variations caused by operational differences [119].

Nevertheless, specific methodologies for batch-to-batch control of these parameters warrant further investigation.

### 4.3. Regulatory Barriers

Due to the diversity of components (polymers, biopharmaceuticals, nanoparticles), the variability of pharmacokinetics, and stringent clinical trial requirements, the approval process for hydrogel-based combination products (such as drug-device combinations) is highly complex. The U.S. FDA and the EU EMA classify these products as combination products, requiring a dual regulatory pathway (e.g., PMA for devices and BLA for biopharmaceuticals), which is estimated to increase approval times by 2–3 years [120] and result in cost increases of 30–50% (i.e., $60 million–$250 million) [121]. Lack of standardized testing protocols for dynamic hydrogels (e.g., degradation rates, immune responses) based platforms for data traceability could streamline regulatory reviews by 40% [107].

### 4.4. Model Limitations

The current TME model oversimplifies mechanical and immunological heterogeneity, limiting its translational relevance. Atomic force microscopy (AFM) shows that the hardness of the tumor invasion front (15–25 kPa) is 3–5 times that of the core region (3–5 kPa) [122], but homogeneous hydrogels such as Matrigel (0.5–50 kPa in vitro and 94–147 kPa in breast cancer in vivo) cannot replicate this gradient [123]. Clinical samples of hepatocellular carcinoma (HCC) show matrix hardening (10–25 kPa) and cellular softening, a contradictory state that in vitro models struggle to simulate, reducing predictive accuracy [124]. Incorporating immune cells and patient-specific organisms brings results closer to clinical outcomes, but scalability remains challenging [125].

## 5. Future Directions: Interdisciplinary Innovations

Innovative approaches aim to overcome these challenges, enhancing dynamic hydrogels’ clinical potential by integrating advanced materials, gene editing, and computational tools.

Hydrogels integrated with biosensors can respond to TME stimuli, enabling real-time drug release. For example, ROS/pH-sensitive hydrogels embedded with nanoscale sensors can release doxorubicin in acidic, high-ROS TME environments. ROS-responsive hydrogels loaded with doxorubicin released over 80% of the drug within five days under 10 mM H_2_O_2_ conditions [102]. Challenges include sensor stability (e.g., untreated PDA sensors exhibited a 60.8% signal decline in PBS and a 75% signal drift in WFE after 30 scans). To improve stability, researchers have tried various methods, such as heat treatment and gel coating, but with limited success [126]; multi-signal coupling (e.g., pH/ROS response) in ROS/pH-sensitive hydrogel therapy for breast cancer enhances treatment efficacy through synergistic effects, including improved drug release efficiency, targeted delivery, regulation of the immune microenvironment, and multifunctionality [127].

### 5.1. Biomimetic Design

The chemo-deformation coupling mechanism refers to a process where a hydrogel senses changes in its chemical environment, such as variations in pH or molecular concentrations, and responds by altering its shape or volume. For example, a hydrogel may shrink in an acidic environment, like that found near a tumor, and expand in a neutral or alkaline one. This behavior acts like a chemical “switch” that triggers physical changes, such as bending or swelling, to perform functions like targeted drug release. In practical terms, this mechanism enables hydrogels to deliver drugs precisely at a tumor site by responding to its acidic conditions.

The nanodrug size adjustment mechanism of cluster missile-like hydrogels describes a sophisticated drug delivery system where a large hydrogel carrier, resembling a cluster bomb, encapsulates multiple smaller nanodrugs. Upon reaching a target site, such as a tumor, the hydrogel disassembles or degrades, releasing these nanodrugs [128]. The key feature is the size adjustment. The large carrier ensures prolonged circulation in the bloodstream and accumulation at the target, while the released nanodrugs, typically 10–100 nanometers in size, can penetrate deeply into tissues. This enhances drug delivery to the core of lesions, improving therapeutic outcomes compared to traditional carriers, which may have penetration efficiencies as low as 20–30% in dense tumor tissues.

Jingge Ma’s team designed a “cluster missile” hydrogel that releases nanodrugs of adjustable size in response to pH/GSH changes in the tumor microenvironment. In the initial stage, small nanoparticles (<50 nm) rapidly penetrate deep into the tumor; in the subsequent stage, large particles (>100 nm) accumulate through a dual reaction within cells. The system also leverages the self-imaging properties of platinum metals, enabling visualization of the treatment process without the need for additional contrast agents. Future developments should explore multi-level penetration strategies (e.g., light-driven nanomotors) and “chemo-deformation” coupling mechanisms that mimic immune cell migration behavior [129].

### 5.2. Synergistic Effects of CRISPR and Hydrogels

Modular Gene Editing: The team led by Academician Tan Weihong has developed a DNA hydrogel containing Cas12a-gRNA complexes, enabling the programming of specific DNA sequences. When target viral RNA (e.g., Ebola virus) is present, the hydrogel network dissociates and releases encapsulated nanoparticles or live cells; Tumor Microenvironment Regulation: Hyaluronic acid/fibrin dual-crosslinked hydrogels can locally deliver CRISPR-Cas9 to knockout the PD-L1 gene in tumor-associated macrophages (TAMs), thereby transforming the immunosuppressive microenvironment into an pro-inflammatory phenotype [130]. Future developments require improving gene editing efficiency (e.g., by regulating the diffusion rate of CRISPR complexes through hydrogel porosity [131]) and developing various logic gate systems (e.g., AND/OR logic targeting multiple tumor markers) [132].

### 5.3. AI-Driven Design

Machine learning is being used to transform traditional hydrogel R&D models, significantly shortening the formulation optimization cycle. For example, an algorithm model based on LightGBM can predict drug release curves, optimize mechanical properties, and establish an automated experimental platform using 17 input features (such as polymer molecular weight, drug loading, and ionic strength of the dissolution medium) [89].

AI-driven approaches are revolutionizing hydrogel design and drug delivery systems by enabling precise optimization of material properties and therapeutic outcomes. For example, a research team at Hokkaido University employed a deep learning model to analyze 24,707 adhesive protein sequences from diverse sources (archaea, bacteria, eukaryotes, viruses, and artificial proteins) [133]. By identifying key amino acid sequence patterns linked to adhesive performance, they designed 180 novel underwater adhesive hydrogels using six functional monomers. Iterative optimization with machine learning, informed by experimental adhesive strength data, led to a high-performing hydrogel variant, R1-max, achieving underwater adhesive strengths exceeding 1 MPa and maintaining adhesion under continuous wave impact [133].

In multi-drug release optimization, reinforcement learning has shown promise. Recently, Polypharmacology Generative Optimization Network (POLYGON) has been developed by combining a variational autoencoder (VAE) and reinforcement learning to design molecules targeting multiple protein receptors simultaneously. POLYGON achieved 81.9% prediction accuracy for high-confidence dual-target activity (IC50 < 1 μM) across a dataset of 100,000 compounds and 1850 targets [134]. When applied to drug-loaded hydrogels, reinforcement learning optimizes release profiles by balancing initial burst release and sustained delivery, achieving prediction errors as low as ±8% for in vitro release rates.

The selection of input parameters for AI models is critical to ensuring accurate predictions in hydrogel design. Key parameters include monomer type and sequence structure, which directly influence hydrogel functionality, such as adhesion or drug-binding affinity, as demonstrated in the Hokkaido study. Polymer molecular weight is another critical parameter, as it affects chain entanglement and mechanical strength, with higher molecular weights (e.g., greater than 50 kilodaltons) enhancing tensile properties. Crosslinking density is often prioritized over molecular weight in load-bearing applications because it directly determines the elastic modulus and swelling behavior of the hydrogel network. The hydrophobic-to-hydrophilic ratio is also considered, as it modulates drug release kinetics and biocompatibility. Environmental conditions, such as pH and temperature, are included because they impact hydrogel swelling and drug diffusion, which are essential for in vivo performance. These parameters are selected based on their well-established influence on structure–property relationships, validated through molecular dynamics simulations and experimental datasets.

For clinical applications, the accuracy of AI predictions must meet stringent requirements to ensure reliability and safety. In surgical sealants, tissue adhesives, or load-bearing implantable hydrogels, predictions of mechanical properties, such as adhesive strength and tensile strength, must have errors within ±10% to ±15% to provide sufficient mechanical support in vivo and prevent postoperative complications, such as tissue dehiscence [133]. For drug-loaded hydrogels, predictions of drug release profiles, including burst release effects and release rates, should maintain errors of ±10% or less in vitro to align with pharmacopeial standards for release testing. In vivo, predictions of hydrogel swelling ratios should have errors of ±20% or less to ensure compatibility with physiological conditions. For critical bioavailability parameters, such as the area under the curve (AUC) and maximum concentration (Cmax), prediction errors should ideally be less than ±8% to ensure therapeutic efficacy. However, these error ranges are not absolute standards. Acceptable margins in actual research and clinical translation depend on specific risk analyses, comparisons with existing standard therapies, and consultations with regulatory agencies to ensure compliance and safety [135].

These advancements demonstrate the transformative potential of AI-driven approaches in hydrogel design and drug delivery systems. Nevertheless, further validation through clinical trials and regulatory approval is necessary to fully realize their practical applicability in medical settings.

## 6. Conclusions

From a chemical perspective, the development of dynamic hydrogels is advancing through innovations in material synthesis and functionalization. Future research will focus on designing hydrogels with enhanced stimuli-responsive properties, such as responsiveness to pH, temperature, or enzymes, to enable precise drug release within the TME. For instance, pH-responsive hydrogels can exploit the acidic tumor microenvironment to trigger controlled drug release, enhancing therapeutic specificity. Novel crosslinking strategies, such as the use of biocompatible agents like genipin or enzyme-based crosslinkers, are being explored to minimize toxicity while maintaining the mechanical integrity of hydrogels. These advancements aim to address batch-to-batch variability in production, a key barrier to large-scale manufacturing, by improving the consistency of hydrogel properties like swelling behavior and drug release kinetics.

The integration of advanced chemical design with technologies such as 3D bioprinting and organoid systems will enable the creation of patient-specific tumor models, improving the ability to predict treatment outcomes. Multi-stimuli-responsive hydrogels, capable of responding to combinations of biochemical cues like reactive oxygen species or glucose levels, will enhance the precision of drug delivery by adapting to the dynamic TME. Additionally, the development of multifunctional hydrogels that co-deliver chemotherapeutic agents, immunomodulators (e.g., IL-12 or anti-PD-1 antibodies), and oxygen carriers will facilitate synergistic therapies, combining multiple treatment modalities to improve efficacy.

Chemically inspired biomimetic designs that replicate the mechanical and biochemical properties of the natural extracellular matrix will improve hydrogel biocompatibility and integration with native tissues. Chemo-deformation coupling mechanisms, inspired by immune cell migration, will enhance tumor penetration by allowing hydrogels to dynamically adjust their shape or porosity in response to chemical gradients in the TME. The integration of CRISPR-based gene editing with hydrogels is another promising direction, where chemically tuned hydrogel structures enhance gene delivery efficiency and specificity for genetic regulation of the TME.

Artificial intelligence (AI) will play a transformative role in advancing hydrogel chemistry by optimizing material formulations and predicting performance. Machine learning algorithms, such as LightGBM, are being utilized to predict drug release profiles and mechanical properties, streamlining the design process through high-throughput screening of chemical compositions. AI-driven platforms will also analyze clinical data to design personalized hydrogel formulations, tailoring treatments to individual patient needs. To support clinical translation, good manufacturing practice (GMP)-compliant production processes will be developed to address scalability and cost challenges, ensuring consistent hydrogel quality. Unified evaluation standards for stimuli responsiveness, degradability, and immunogenicity will be established to facilitate regulatory approval, ensuring hydrogels meet clinical safety and efficacy requirements.

## Figures and Tables

**Table 4:** Application of bioprinting and microfluidic technology in tumor model construction.

Technology	Hydrogel Type	Application	Outcome	Reference
Bioprinting	GelMA	3D tumor modeling	Replicates ECM stiffness	[79]
Bio-3D Printing	GelMA	Chemotherapy response prediction	85% chemotherapy response accuracy	[81]
Immune-Tumor Co-culture	Immune cell-loaded bioinks	Simulate immune-tumor interactions	70% fidelity to in vivo conditions	[82]
Vascular network modeling	Bioinks with cells/biomaterials	Tumor angiogenesis study	Enables creation of perfusable vascular networks resembling in vivo complexity	[89]
Microfluidics	Alginate-Gelatin	Angiogenesis simulation	75% vascular structural similarity	[94]
Microfluidics-Organoid	GelMA	Metastatic invasion modeling	90% accuracy with colorectal cancer organoids	[95]

## Abbreviations

The following abbreviations are used in this manuscript:

TME	tumor microenvironment
ECM	extracellular matrix
CAFs	Cancer-associated fibroblasts
MMP	matrix metalloproteinases
LOX	lysyl oxidase
Treg	regulatory T cells
IL-2	interleukin-2
MDSCs	myeloid-derived suppressor cells
Arg-1	arginase-1
iNOS	inducible nitric oxide synthase
CSCs	cancer stem cells
CRC	colorectal cancer
EMT	epithelial-to-mesenchymal transition
MET	mesenchymal-to-epithelial transition
ECs	endothelial-like cells
anti-PD-1	anti-programmed death-1
ALG-βCD	in situ formed βCD-modified alginate
Ad	adamantane
VEGF	vascular endothelial growth factor
HA	hyaluronic acid
PEG	Polyethylene glycol
Ad-aPD1	adamantane-modified anti-PD-1 antibodies
NLCs	nanostructured lipid carriers
PFCs	perfluorocarbons
PDT	photodynamic therapy
TPL	triptolide
CA IX	carbonic anhydrase IX
PepABS-py	Self-assembled short peptide
GSH	glutathione
GnRH/LHRH	Gonadotropin-releasing hormone/Luteinizing Hormone-Releasing Hormone
ROS	Reactive Oxygen Species
GelMA	Gelatin acryloyl
CAD	computer-aided design
PTT	photothermal therapy
IRE1α	Inhibition of inositol-requiring enzyme 1
FlaB	flagellar protein B
VEGFR	vascular endothelial growth factor receptors
ICB	immune checkpoint blockade
DC	dendritic cell
TLR	Toll-like receptor
TAMs	tumor-associated macrophages
α-PDL1/PEIGel	The PVA-PEI hydrogel
CAR-T	Chimeric Antigen Receptor T-Cell
GMP	Good Manufacturing Practice
AFM	Atomic force microscopy
HCC	hepatocellular carcinoma
VAE	variational autoencoder
AUC	area under the curve
POLYGON	Polypharmacology Generative Optimization Network
